# Seminal Fluid-Mediated Inflammation in Physiology and Pathology of the Female Reproductive Tract

**DOI:** 10.1155/2016/9707252

**Published:** 2016-07-03

**Authors:** Anthonio O. Adefuye, Henry A. Adeola, Kurt J. Sales, Arieh A. Katz

**Affiliations:** ^1^Division of Immunology, Department of Pathology, University of Cape Town, Faculty of Health Sciences, Observatory 7925, South Africa; ^2^International Centre for Genetic Engineering and Biotechnology and Department of Integrative Biomedical Sciences, Faculty of Health Sciences, University of Cape Town, Observatory 7925, South Africa; ^3^MRC/UCT Receptor Biology Research Unit, Department of Integrative Biomedical Sciences and Institute of Infectious Disease and Molecular Medicine, Faculty of Health Sciences, University of Cape Town, Observatory 7925, South Africa; ^4^SAMRC Gynaecology Cancer Research Centre, Faculty of Health Sciences, University of Cape Town, Observatory 7925, South Africa

## Abstract

Inflammation is a multifaceted process involving a host of resident and recruited immune cells that eliminate the insult or injury and initiate tissue repair. In the female reproductive tract (FMRT), inflammation-mediated alterations in epithelial, vascular, and immune functions are important components of complex physiological processes and many local and systemic pathologies. It is well established that intracoital and postcoital function of seminal fluid (SF) goes beyond nutritive support for the spermatozoa cells. SF, in particular, the inflammatory bioactive lipids, and prostaglandins present in vast quantities in SF, have a role in localized immune modulation and regulation of pathways that can exacerbate inflammation in the FMRT. In sexually active women SF-mediated inflammation has been implicated in physiologic processes such as ovulation, implantation, and parturition while also enhancing tumorigenesis and susceptibility to infection. This review highlights the molecular mechanism by which SF regulates inflammatory pathways in the FMRT and how alterations in these pathways contribute to physiology and pathology of the female reproductive function. In addition, based on findings from TaqMan® 96-Well Plate Arrays, on neoplastic cervical cells treated with SF, we discuss new findings on the role of SF as a potent driver of inflammatory and tumorigenic pathways in the cervix.

## 1. Introduction

Characteristically regarded as response to tissue injury or pathogenic insult, inflammation is a multifaceted process that involves a host of resident and recruited immune cell types working together to promote the elimination of insult or injury and initiate tissue repair [1].

In the female reproductive tract (FMRT), complex physiological processes such as menstruation, ovulation, parturition, and implantation have been shown to display hallmark of inflammation [1]. These reproductive events are associated with the upregulation of inflammatory mediators including cytokines, growth factors, and lipid mediators within the host [2]. Similarly, it is well recognized and widely accepted that inflammation-mediated alteration in epithelial, vascular, and immune functions within the female reproductive tract are important components of many local and systemic pathologies including cervical cancer, endometrial cancer, pelvic inflammatory disease, and HIV infection [3]. In addition to endogenous physiologic response, inflammation within the female genital tract can be mediated by a variety of exogenous effectors, including exposure to seminal fluid (SF) [4, 5].

It is well established that intracoital and postcoital function of SF goes beyond nutritive support for the spermatozoa cells. Consisting of a complex mixture of molecules including glycoproteins, cytokines, growth factors, and prostaglandins [6], SF can mount inflammatory responses in the female reproductive tract [5, 7]. These SF-mediated inflammatory responses have been suggested to impact on both physiologic [8] and pathologic conditions within the FMRT. In this review, we discuss the molecular mechanism by which SF regulates inflammation and inflammatory pathways in the female reproductive tract and how alterations in these pathways contribute to physiology and pathology of the female reproductive function.

## 2. Immune Response and Inflammation in the FMRT

Inflammation is an integral part of the immune system response to infection or tissue injury [9]. Anatomical structures within the FMRT are divided into two distinct groups, namely, the sterile upper FMRT (the fallopian tubes, uterus, and endocervix) and the nonsterile lower FMRT (ectocervix and vagina) [10]. Consequent of its close proximity to the rectum, the lower FMRT is highly prone to secondary bacterial contamination [11] and as such requires an efficient immune system to protect the host from overwhelming infection.

Constituents of the innate immune system such as the pattern recognition toll-like receptors (TLRs), natural antimicrobial peptides (NAPs), defensins, and the complement system all work in synergy to protect the FMRT from exogenous threats [12]. TLRs are ubiquitously expressed in the endometrium, cervix, fallopian tubes, and epithelial cells [13]. TLRs recognize pathogen-associated molecular peptides, microbial-associated molecular peptides, and danger-associated molecular peptides and respond by mediating increased cytokine and chemokine production leading to increased chemotaxis of monocytes and neutrophils into surrounding tissue.

NAPs including secretory leukocyte protease inhibitor (SLPI) and elafin are found in highest concentration within the cervical mucus and vagina, where they prevent host tissue damage by inhibiting proteases released by Gram-negative and Gram-positive bacteria [10, 12, 14]. Human defensins (*α* and *β*) are small cationic proteins that have antibacterial, antifungal, and antiviral properties important for host protection [10]. *α*-defensins are found in neutrophils and epithelial surfaces as human neutrophil peptides 1–4 and human *α*-defensins 5 and 6, respectively, while *β*-defensins are found primarily on epithelial surfaces as human *β*-defensins (HBDs) 1–6 [10, 14]. Defensins can be constitutively expressed or induced after inflammatory or infectious stimulus [10, 14]. The chemoattractant properties of *β*-defensins facilitate interaction between the innate and adaptive immune responses [14].

Cell-mediated immune responses protect the female from sexually transmitted intracellular disease causing agents. Immune cells are differentially distributed in each organ of the FMRT [15]. CD3^+^ T cells are distributed throughout the FMRT, while B cells are rare [15]. However, unlike the peripheral blood the FMRT consist of more CD8^+^ and less CD4^+^ T cells [15]. In addition, antigen-presenting cells (APCs) found in the FMRT have been shown to constitutively express MHC (major histocompatibility complex) class II molecules [16].

However, while eliminating threatening sexually transmitted and environmental pathogens, the mucosal immune system of the FMRT is uniquely adapted to facilitate specialized physiological functions such as ovulation, menstruation, implantation, pregnancy, and parturition [10].

## 3. Physiologic Inflammation and FMRT

Physiologic inflammation is usually self-limiting because production of proinflammatory cytokines also gives way to anti-inflammatory cytokine secretion as the healing progresses [17]. In the female reproductive tract, injury and tissue remodeling orchestrated by physiological process such as ovulation, menstruation, implantation, and parturition triggers inflammatory cascade [1].

### 3.1. Ovulation

Ovulation is a complex physiologic process that involves series of biochemical and biophysical events that ultimately lead to the rupture of the preovulatory follicle and the release of the maternal germ cell [18]. Ovulatory process is initiated by luteinizing hormone (LH) surge [19]. This process displays all the hallmark of acute, physiologic (self-controlled) inflammatory reaction, including expression of inflammatory molecules (PGs, LTs, histamine, and cytokines), leukocyte extravasation, edema, hyperaemia, and induction of proteolytic and collagenolytic activities [20, 21]. Furthermore, the expression of innate immune cell-related surveillance proteins (toll-like receptors 2 and 4) by the ovarian granulosa cells and cumulus cells during ovulation thus suggest a role for the innate immune system in this process [22].

### 3.2. Menstruation

Menstruation results from partial breakdown of the functional layer of the endometrium accompanied by shedding of cell debris and uterine bleeding that occurs following the fall in progesterone level resulting from the demise of the corpus luteum at the end of a normal reproductive cycle [23]. Menstruation involves a complex series of events parallel to those of inflammatory response. Within the endometrial epithelial cells, progesterone withdrawal releases NF-*κ*B from its inhibition by I*κ*B leading to the induction of inflammatory gene, including COX-2, MMP-9, CXCL8, CCL5, Mn-SOD, CCL1, CCL3, IL-6, IL-1*β*, CXCL1, GM-CSF, TNF-*α*, and CXC chemokines resulting in an influx of inflammatory cells [24, 25]. These proinflammatory mediators and inflammatory cells induce cascade of degradative enzymes, particularly matrix metalloproteinase in the endometrial epithelial cells, inducing both their production and activation leading to rapid breakdown of the extracellular matrix supporting the tissue.

### 3.3. Implantation

Embryo implantation starts with blastocyst apposition to the uterine endometrium, followed by its attachment to the endometrial surface epithelium [26]. This process bears a resemblance to “an open wound” requiring a strong inflammatory response [27]. The blastocyst breaks through the uterine epithelial lining to implant, thus creating an arena of invading, dying, and repairing cells [26]. Substantial amounts of proinflammatory Th1 cells and cytokines including TNF*α*, IL-8, and IL-6 characterize early implantation [28, 29]. In addition, elevated levels of PGs are found in areas of increased endometrial vascular permeability associated with initiation of implantation, suggesting that PGs play an important role during implantation [30]. These potent inflammatory mediators can be secreted by the endometrial cells themselves as well as by infiltrating immune cells recruited to the site of implantation [27]. Similarly, secretion of anti-inflammatory mediators such as IL-10 and adiponectin helps prevent excessive inflammation, suggesting that regulation of inflammation during implantation is a sequential event in which proinflammation is followed by anti-inflammation or both occurring in continual balance to attain physiologic state.

### 3.4. Parturition

Emerging evidence shows that normal parturition is characterized by massive influx of immune cells including neutrophils and macrophages into the myometrium and the cervix [31, 32]. The invading leukocytes as well as the myometrium, cervix and foetal membranes collectively release proinflammatory mediators such as cytokines and PGs to promote myometrial contractility via the upregulation of TNF-*α* and IL-1*β* whose effect on the myometrium is similar to that of oxytocin, by inducing COX-2 expression and the production of PGE_2_ in the myometrial cells [33, 34]. In addition, these mediators promote cervical ripening and membrane rupture via the activation of MMPs, cathepsin S, and COX-2 and inhibition of tissue inhibitor of metalloproteinase- (TIMP-) 2 [35–38].

## 4. Inflammation and Pathology of FMRT

Deregulation of any of the mediating factors in the inflammatory cascade can lead to abnormalities and ultimately pathogenesis.

### 4.1. Pelvic Inflammatory Disease (PID)

PID is an inflammatory condition of the FMRT (cervix, uterus, fallopian tubes and adjacent pelvis structures) caused by sexually transmitted microorganism including* Chlamydia trachomatis* and* Neisseria gonorrhea *[39]. Infection of the epithelial cells by these microorganisms is characterized by secretion of proinflammatory cytokines responsible for disease progression [40, 41]. The innate immune system remains the first line of defense against pathogenic infection and relies on conserved family of pattern recognition receptors (PPRs) including toll-like receptor (TLR) family [42] for pathogen recognition [43, 44]. TLRs are expressed in the FMRT [44]. After binding to bacterial ligand, TLR signaling mediates the induction of proinflammatory cytokine and chemokine genes and priming of the adaptive immune system that leads to pathogen elimination [45]. However, increased or persistent TLR signaling can prolong the inflammatory response [46].

### 4.2. FMRT Cancers

The inverse association between the long-term use of NSAID and reduced risk of several cancers supports the link between inflammation and cancer [47, 48]. Similarly, several epidemiological studies have documented a positive relationship between local tissue inflammation and risk of cancer development [49]. Although the detailed molecular mechanism of inflammation-mediated cancer still remains elusive, it is established that proinflammatory cytokines such as TNF-*α* and IL-1*α* produced by inflamed local tissue and infiltrating immune cells play a vital role [17] via the induction of rapid cell division and production of free radicals that subsequently cause DNA damage [50]. In the context of FMRT malignancies, inflammation can contribute to the initiation and progression of neoplastic conditions via the release of cytokines and growth factors to facilitate immune cell recruitment, cell proliferation, angiogenesis, and sustained tumor growth [1]. In the FMRT, ovarian, endometrial, and cervical cancer are the most common gynecological malignancy encountered [51].

Epithelial ovarian cancer (EOC) is a highly lethal gynecological cancer characterized by overall poor prognosis over the past few decades [52]. Although the genetic events within the neoplastic cells themselves are crucial for disease development, the most important hypothesis regarding EOC carcinogenesis remains the ovulation theory which relates the risk of cancer development to incessant ovulation [52, 53]. Indeed, the protective effect of oral contraceptives (OCs) has been subsequently reported in several studies [54]. The ovulatory process and repair steps following the release of the ovum is characterized by recruitment of immune cells to the wounded epithelial surface and secretions of enormous amount of cytokines, chemokines, PGs, bioactive eicosanoids, plasminogen activation factor, collagenases, and various growth factors [55], reminiscent of a proinflammatory network. Similarly, components of proinflammatory pathway signaling including ILs/cytokines, free radicals, NF-*κ*B, STAT-3, iNOS, COX-2/PGs, and VEGF have been shown to promote EOC genesis, growth, and progression [56, 57]. COX-2 was found to be upregulated in nonmucinous ovarian cancers, and its expression was correlated with poorer prognosis [58]. Continual exposure of the ovarian surface epithelium (OSE) adjacent to the site of ovulation to this inflammatory and oxidative milieu results in an increased risk of neoplastic transformation. Consistent with these findings, it has been shown that patients with chronic NSAID use have a reduced risk of EOC [59].

Circumstantial evidence linking inflammation and endometrial neoplasia has been described [49]. Given the inflammatory components of menstruation as described earlier, these factors could bear on the initiation and progression of endometrial neoplasia [60]. Studies on endometrial tissue explant shows that NF-*κ*B is overtly expressed in endometrial hyperplasia and endometrial carcinoma and notably decreased NF-*κ*B expression also coincides with an increase in apoptosis in low-grade cancer [61]. Besides its role as a major transcription factor for numerous proinflammatory genes, NF-*κ*B induces the expression of COX-2 in endometrial carcinoma [62] where it enhances the production of PGE_2_ in both the malignant and adjacent endometrial stromal cells [63]. Within the neoplastic endometrium, elevated COX-2 and PGE_2_ can facilitate angiogenesis [64], increase cell proliferation, decrease apoptosis [65], and facilitate tissue invasion [66] leading to increased tumor aggressiveness. Several prospective cohort studies and* in vitro* experiments have shown that the use of aspirin is associated with reduced risk of endometrial cancer and inhibition of endometrial cancer cell growth [47] suggesting that endometrial cancer is inflammatory-dependent.

Cervical cancer is a chronic inflammatory disease and one of the leading causes of cancer-related death worldwide with a higher incidence rate reported in underdeveloped countries [67]. It is well established that persistent infection with high-risk HPV is crucial to disease pathogenesis [68]. However, only a subset of women infected with high-risk HPV will proceed to develop invasive cervical cancer, thus suggesting that other cofactors must be present for the development of malignancy [69]. Studies have reported an association between the level of cervical inflammation and the development of high grade cervical neoplasia [70] or invasive cervical cancer [71]. It has been reported that cervical inflammation but not the actual diagnosis of a specific sexually transmitted infection is associated with the development of squamous intraepithelial lesions within the cervix [72]. Direct links between increased proinflammatory cytokine levels in patients and increasing grade of cervical intraepithelial neoplasia and invasive cervical cancer have been established [73]. Similarly, proinflammatory COX-PGs axis has been shown to be elevated in cervical cancer [74].

## 5. SF a Potent Inflammatory Mediator in the FMRT

SF is a complex endogenous fluid comprising secretions of male accessory sexual glands (cowper's and littre glands, prostate, and the seminal vesicles) that provides nutritive support to the mammalian spermatozoa [75]. Secretions from each of these reproductive organs are biochemically distinct and, on mixing as occurs at ejaculation, give rise to the complex biochemical nature of the SF. Exposure of the FMRT to SF during coitus has been shown to elicit substantial changes in the leukocyte populations within the cervix, initiating a reaction reminiscent of inflammatory response with effects that penetrate through the stratified epithelial layer and deep into the stroma of the ectocervix [5]. This finding is supported by the lack of an observed inflammatory response in the absence of coitus or with condom-protected coitus [5]. After coitus, the degree at which SF normally activates the secretion of proinflammatory components in any compartment of the female reproductive tract is poorly understood. However, it has been established that influx of immune cells expands inducible regulatory T cell population, promoting immune tolerance there by preparing the reproductive tract for conception [5, 76, 77].

### 5.1. SF PGE_2_ Potent Proinflammatory Mediator in the FMRT

SF is known to contain an enormous diversity of antigenically distinct molecules [78]. However, it is the highly expressed levels of prostaglandins found in human SF [79] that have attracted much interest of late. Of the PGs present in SF, PGE_2_ has been identified as one of the predominant types detected [80, 81]. PGE_2_ is a strong chemotactic agent for neutrophils [82]. Over the years, studies have shown that overt expression of PGE_2_ and its signaling is found in numerous disorders including cervical cancer [83, 84] and women infected with HIV and HPV [85, 86]. It has been shown that PGE_2_ potentiates the chronic inflammatory response seen in these diseases, leading to greater tumorigenesis (cervical cancer) [84] and enhanced viral replication (HIV infection) [87]. Study by Joseph et al. identified PGE_2_ present abundantly in SF as the main constituent responsible for SF-mediated inflammatory effects in vaginal cells [88].

Recently, we identified SF-PGE_2_ as the main constituent responsible for SF-mediated regulation of pleotropic proinflammatory cytokine and HIV chemokine coreceptor in normal and neoplastic cervix [89, 90].

## 6. SF-Mediated Inflammation and Physiology of the FMRT

### 6.1. SF and Ovulation

Since the ovulatory process has been likened to an inflammatory reaction which includes immune cell infiltration of the tissue of the Graafian follicles [21], it has been speculated that SF could mediate leukocyte trafficking from the uterus to the ovary and that migrating leukocytes can serve as vector in augmenting ovulation [82]. In mammals, studies have shown that SF advance the anticipated time of ovulation by significant number of hours [91]. Similarly, it has been shown that SF of certain mammals contain ovulation-inducing factors that induce a surge in circulating concentrations of LH and induced an ovulatory and luteotropic response [92].

Although the mechanism and route of signal transduction from the uterus to the ovary are unknown, it is believed that signaling pathways could involve locally induced cytokines such as GM-CSF and TNF-*α* secreted by uterine epithelial cells after SF stimulation [4].

Several* in vitro* and* ex vivo* studies have shown that TNF-*α* can induce ovulation or trigger events leading to ovulation [93]. These mediators may reach the ovarian stroma and preovulatory follicles via the lymphatic ducts and a countercurrent transfer system from the uterine vein to the uteroovarian artery and bind to receptors expressed on the surface of the ovarian cells [82]. While the effects of SF on ovulation have not been documented in humans, it is plausible that it act in similar manner to enhance the ovulatory process.

### 6.2. SF, Implantation and Parturition

After fertilization, the survival of the semiallogeneic zygote depends on adaptations and immune tolerance in maternal innate and adaptive immune system [94]. This tolerance is mediated in part by a unique subpopulation of T cells, regulatory T (Tregs) cells [95]. Tregs control immunologic self-tolerance by suppressing the generation of effector T cells (Teff) via an indoleamine-2,3-dioxygenase regulated pathway [96]. Robertson et al. demonstrated that exposure to SF at mating in mice promotes a state of immune tolerance to paternal alloantigens that may facilitate maternal acceptance of the conceptus at implantation, and the effects of SF are likely to be mediated by expansion of the Tregs cell pool [76]. It is well established that SF contains potent immune-regulatory molecules including TGF*β* and PGE-related prostaglandins, particularly 19OH-PGE_1_ and 19OH-PGE_2_ [76]. These molecules facilitate the unique Tregs cell-inducing properties of SF by inducing naıve CD4^+^ CD25^−^ T cells to differentiate into suppressor T cells expressing Foxp3 [97, 98]; these molecules facilitate the unique Tregs cell-inducing properties of SF. These findings suggest a role for SF in regulating maternal immune tolerance, a key factor required for successful implantation.

The role of coitus in cervical ripening and induction of labour has been extensively reviewed in [99]. It was suggested that coitus may stimulate cervical ripening and the onset of labour in part by the direct action of PGs present in SF [99]. However, there is insufficient data at present to confirm these findings.

## 7. SF-Mediated Inflammation and Pathology of the FMRT

### 7.1. SF Hypersensitivity (Seminal Plasma Hypersensitivity)

As discussed earlier, the human SF contains diverse range of antigenically distinct molecules including the prostate-specific antigen (PSA), a 33-34 kDa glycoprotein with serine-protease activity [14, 100, 101]. Exposure of the FMRT to this SF-derived glycoprotein has been the main aetiological factor implicated in SF hypersensitivity [101]. SF hypersensitivity is an IgE mediated type I hypersensitivity reaction characterized by a spectrum of clinical symptoms manifesting as either systemic and/or localized reactions after exposure to specific protein components in SF [5]. However, unlike typical allergic reactions SF hypersensitivity rarely has a heritable/familial component [102]. It has been postulated that SF proteases such as PSA can cause degradation of vagina mucosal tight junctions, leading to the activation of protease activated receptor- (PAR-) 2 on the vagina epithelial cells and release of proinflammatory cytokines and localized inflammation [101]. In addition, SF-PGE_2_ has been implicated in the pathophysiology of localized SF hypersensitivity. This is supported by findings by Ghosh and Bernstein, wherein women with localized SF hypersensitivity experience attenuation of their symptoms following ingestion of nonsteroidal anti-inflammatory agent prior to unprotected intercourse [101]. This pathophysiology is consistent with histopathological findings that reveal a localized nonspecific inflammation and inconsistent with a typical allergic IgE-mediated reaction [103]. However, further investigation of the mechanism(s) related to localized SF hypersensitivity is warranted.

### 7.2. SF and Endometrial Tumorigenesis

Emerging evidence suggests that constituents of SF can travel into the endometrium and regulate gene expression [104]. Thus, suggesting that, in sexually active women, endometrial pathologies may be enhanced following exposure to SF [105]. This was confirmed in a study by Sales et al. where SF was shown to modulate neoplastic endometrial cell function. Sales and colleagues demonstrated that SF, acting via EP2-EGFR-ERK signal pathways upregulated the expression of the potent mitogenic/proangiogenic gene, fibroblast growth factor 2 (FGF2), in the neoplastic endometrium [106].

### 7.3. SF and Cervical Tumorigenesis

Central to the role of SF in augmenting cervical tumorigenesis is its ability to regulate the inflammatory processes in the cancer milieu. Lipid components of SF can be reabsorbed into the vaginal fornix or local tissues [107], where they can regulate tissue remodeling processes in an autocrine or paracrine manner [5]. We and others have demonstrated the proinflammatory role of SF on neoplastic cervical epithelium. These studies reveal that SF can promote cancer cell proliferation by inducing the expression of angiogenic genes and pleotropic proinflammatory cytokines/chemokines via the activation of pathways including COX-PGs, EGFR-ERK 1/2, NF-*κ*B, and EP2-EGFR-PI3kinase-AKT pathways [89, 108–110]. Discovery of these pathways suggests that SF can regulate a wide range of inflammatory pathways to augment cervical tumorigenesis. Hence, with the aim of investigating gene arrays of inflammatory pathways that can be regulated by SF in neoplastic cervical epithelial cells, cervical adenocarcinoma cells (HeLa) were treated with serum-free media containing SF at a dilution of 1 : 50 or serum-free media with PBS (control) for 8 hrs (*n* = 5 individual experiment done in duplicate). The 5 individual experiments were pooled together, RNA extracted, and the synthesized cDNA subjected to Real-Time RT-PCR using TaqMan Plate Array (Human inflammation and human chemokines) (Applied Biosystems, USA) [90] on a Bio-Rad CFX96*™* quantitative RT-PCR system. Relative expression was calculated using the comparative C_t_ method and arrays were normalized for RNA loading using 18s ribosomal RNA, glyceraldehyde-3-phosphate dehydrogenase (GAPDH), hypoxanthine phosphoribosyltransferase 1 (HPRT1), and glucuronidase beta (GUSB) as internal controls. In a separate experiment, validation of the TaqMan 96 array assay was done by comparing mRNA expression of selected genes (IL-1*α*, IL-8, IL-12*α*, PTGIR, PTGFR, and CXCR4) in HeLa S3 cells treated with SF (1 : 50) or PBS (control) for 4, 8, 16, and 24 hrs, respectively, and Real-Time RT-PCR done using SYBR Green on an Eco*™* Real-Time PCR system (Illumina*™*).

We found that SF regulates components of eicosanoid signaling (cyclooxygenase and lipoxygenase), kallikrein-kinin-bradykinin receptor signaling, toll-like receptor-2 (TLR2) signaling and chemokine/cytokine signaling in neoplastic cervical epithelial cells (unpublished data) (Tables 1, 2, and 3).

Eicosanoids, including prostaglandins and leukotrienes, are biologically active lipids that have extensively been implicated in inflammation and cancer [111]. COX-1 expression has been shown to be upregulated in numerous human neoplasia [112]. SF-mediated regulation of COX-1 (2.43-fold induction) in the array is in agreement with a similar study by Sutherland et al. [110]. A major metabolite of COX enzyme is PGE_2_. PGE_2_ biosynthesis and signaling are significantly elevated in cervical cancer where it modulates tumor cell proliferation, differentiation, and apoptosis [109, 110, 113]. Hence, in sexually active women exposure to SF can lead to the activation of the COX-PGs signaling pathway consequent of COX-1 induction. Activated COX-PGs pathway can then promote PGE_2_-EP signaling, which can act in similar manner to enhance cervical inflammation and tumorigenesis. In addition, SF-mediated regulation of COX-1 enzyme has recently been implicated in the upregulation of CCR5, an HIV chemokine coreceptor in the cervix [90]. Increase in HIV susceptibility can then further potentiate cervical tumorigenesis.

Similarly, it was observed that SF could also potentially regulate inflammation and tumor progression by inhibiting negative feedback regulators or inhibitory/anti-inflammatory pathways. The release of AA by phospholipase A_2_ is negatively regulated by annexin (encoded by* ANXA 1–5*), a group of cellular proteins known to inhibit cPLA_2_ activity [114]. The gene array showed that SF inhibited annexin 1, 3, and 5 expressions by 6.66-fold, 3.44-fold, and >100-fold reduction, respectively (Table 1). By inhibiting annexin expression and upregulating phospholipase A_2_ (3-fold induction) expression (Table 1), SF ensures steady and continuous release of AA metabolite into the cascade for PGH_2_ synthesis, thereby driving inflammatory pathways and potentially enhancing tumor progression, by enhancing biosynthesis of PGE_2_, PGF_2*α*_, and PGI_2_ [111, 115, 116].

Furthermore, in addition to PGE_2_ production, inhibition of pathways that metabolize PGE_2_ can also prolong inflammation by enhancing ligand receptor interaction. PGE_2_ is metabolized through oxidation of its 15(S)-hydroxyl group by NAD^+^-linked 15-hydroxyprostaglandin dehydrogenase (15-PGDH, encoded by HPGD) to inactive 15-keto products [117–119]. 15-PGDH is a cytosolic enzyme that has been reported to act as bladder, breast, gastric, lung, and colorectal tumor suppressor [120–125]. In immunodeficient mice, 15-PGDH inhibits the development of murine intestinal neoplasia [125, 126]. These findings supported with the fact that 15-PGDH expression is abolished in various cancers [118, 127], emphasizing the oncogenic potential of the PG biosynthesis pathway. In the array, SF suppressed HPGD expression (3.12-fold inhibition) (Table 1). This can lead to reduction in PGE_2_ degradation and increased steady state levels of PGE_2_ resulting in enhanced cervical tumorigenesis.

Leukotrienes are powerful lipid mediators of inflammation in various acute and chronic inflammatory diseases [128, 129]. Compared with PGs, much less is known about the role of proinflammatory leukotrienes in cancer [111]. However, emerging data suggest that leukotrienes can play vital roles in carcinogenesis [111]. Data from the gene array shows that SF induces the expression of leukotriene C_4_ synthase (LTC_4_S) (10-fold induction) (Table 1), an enzyme that catalyzes the conjugation of leukotriene A_4_ (LTA_4_) to form leukotriene C_4_ (LTC_4_), the parent compound of the cysteinyl leukotrienes (CysLTs) (LTC_4_, LTD_4_, and LTE_4_) [130]. CysLTs exert their biological activity by binding to two distinct CysLTs receptor (CysLT1 and CysLT2) [131, 132], with higher binding affinity to CysLT1 than CysLT2 [111]. CysLTs-CysLT1 signaling has been suggested to play an important role in carcinogenesis. In human colorectal and prostate cancer, CysLTs-CysLT1 signaling has been shown to mediate proliferation and inhibit apoptosis, thus conferring poor prognosis [133, 134]. It is likely that CysLTs-CysLT1 signaling could regulate cervical cancer via similar mechanisms. Taken together, these findings suggests that SF can potentially enhance cervical tumorigenesis by not only directly enhancing proinflammatory and protumorigenic lipid signaling pathway (COX-PGE_2_ and LTC_4_S-CysLTs,) but also indirectly concomitantly inhibiting the expression of enzyme systems that metabolize ligands of these pathways (HPGD), thereby driving inflammation forward.

Kallikrein-related peptidases (KLKs) belong to a subgroup of secreted serine proteases [135] that catalyzes several physiological processes within the human body [136]. SF induces the expression of several KLKs (KLK 2, 3, 14, and B1) and only marginally reduces the expression of KLK1 by 1.56-fold reduction as seen in the gene array (Table 2). Emerging evidences designate a possible role for KLKs in inflammation and various cancer processes [136]. KLKs have been shown to mediate cancer cell proliferation and tumor growth mainly via insulin-like growth factors (IGFs) [135]. Activation of KLKs by inflammatory stimuli such as PGs in SF can result in the generation of biologically active kinins by limited proteolysis of kininogens [137, 138]. Bradykinins are a group of pluripotent peptides implicated in various pathophysiological events [139]. This suggests that continuous exposure of cervical epithelial cells to SF can activate KLKs-kinin-BDKR1 signaling pathways to mediate chronic inflammatory reactions and augment tumor progression within the cervix.

Toll-like receptor- (TLR2-) 2 belongs to the family of type I transmembrane receptors structurally characterized by extracellular leucine rich repeats (LRRs) and an intracellular toll/IL-1 receptor (TIR) signaling domain [140]. TLR2 is activated by a wide range of pathogen-associated molecular patterns (PAMPs) including lipoproteins and bacterial lipopolysaccharides (LPS) [141]. Hence, it is very feasible that SF-induced TLR2 (88-fold induction) can be activated by lipoproteins that might be present within SF. Activated TLR2 can then recruit SF-induced MyD88 (9.41-fold induction) and NF-*κ*B (2.32-fold induction) (Table 3) to activate the TLR2-MyD88-NF-*κ*B signaling pathway. Translocated NF-*κ*B can then mediate the release of proinflammatory and tumorigenic genes from the neoplastic cells themselves to augment cervical tumorigenesis. Consistent with this, Xie et al. showed that the activation of TLR2 in breast cancer cells significantly promote cellular invasion via the activation of NF-*κ*B [142]. Furthermore, in sexually active women, STIs such as gonorrhea are known cause of chronic cervicitis, a persistent inflammation of the cervix, which has been linked to cervical cancer [143, 144]. It is plausible that the bacterial LPS present within infective SF can bind to the induced TLR2 to activate the TLR2-MyD88-NF-*κ*B signaling pathway, releasing proinflammatory modulators to mediate cervical inflammation.

Recently chemokines and their receptors have been identified as key mediators of chronic inflammation, which play an important role in the pathogenesis and progression of various human cancers including cancer of the cervix [145–148]. SF regulated the expression of chemokines CCL2, CCL5, CXCL1, CXCL2, CXCL3, CXCL8 (IL-8), and CXCL11 (Table 3) all of which play vital role in cervical cancer inflammation and tumorigenesis.

CCL2 (MCP-1) and RANTES (CCL5) are major determinants of macrophage and lymphocyte infiltration [149]. Hence, SF-mediated induction of CCL2 (MCP-1) and CCL5 (RANTES) (4.43- and 5.24-fold induction, resp.) (Table 3) suggests a role for both MCP-1 and RANTES in the characteristic postcoital inflammatory response described by Sharkey et al. [5]. The SF-mediated induction of CCL2 and CCL5 (4.43- and 5.24-fold induction) is in agreement with similar findings by Chen et al. wherein SF was shown to induce the expression of CCL2 and CCL5 in endometrial epithelial cells* in vitro* [150]. In addition, Schjenken et al. showed that SF mediated a 3.06-fold induction of CCL2 in the endometrium of CBAF1 female rats 8 hours after mating with an intact Balb/c male rat [151]. CCL2 and CCL5 have been shown to modulate cell migration, invasion, and metastasis in several cancer cells [152]. It is likely that SF-induced CCL2 and CCL5 can act in similar manner to enhance cervical cancer cell migration, invasion, and metastasis.

Induction of CXCL1 (12.37-fold induction) and CXCL8 (38.9-fold induction) by SF as seen herein is in agreement with a similar study by Sales et al., where it was shown that SF induces the expression of CXCL1 and CXCL8 in HeLa cells and regulates vascular function* in vitro* [113]. Likewise, Chen and colleagues showed that SF mediated 6.5-fold and 14.4-fold induction of CXCL1 and CXCL8, respectively, in endometrial stromal fibroblast cells* in vitro* [150]. Furthermore, Wang et al. showed that PGE_2_ induces the expression of CXCL1 in human colorectal cancer cells and that expressed CXCL1 then induces microvascular endothelial cell migration and tube formation* in vitro* [153]. It is therefore very probable that the copious PGE_2_ found in SF [80] can mediate CXCL1 induction in neoplastic cervical cancer cells. Thus, suggesting that exposure of neoplastic cervical epithelium to SF can lead to increased tumor angiogenesis and invasiveness/metastasis consequent of CXCL1 and IL-8 expression.

Similarly, findings from the array showed that SF regulates the expression of pleotropic proinflammatory cytokines IL-1*α*, TNF, and IL-6 and components of their respective signaling cascade in neoplastic epithelial cells (Table 3). This is in agreement with a study by Adefuye et al. where it was shown that SF induced the expression of IL-1*α* in both normal and neoplastic cervix via EP2-EGFR-PI3kinase-AKT signaling [3]. Furthermore, Castrilli et al. reported that IL-1*α* promotes the growth of both normal and neoplastic human cervical epithelial cells* in vitro* [154], thus suggesting a similar role for IL-1*α in vivo*. SF induction of IL-6 is in keeping with similar study by Sutherland et al. [110]. SF-mediated downregulation of suppressor of cytokine signaling- (SOCS-) 5 (Table 3) suggests that SF augments proinflammatory cytokine signaling within the cervical cancer microenvironment to enhance disease progression.

Induction of immune modulatory cytokines IL-12*α* and IL-18 by SF (Table 3) suggests a role for SF in the regulation of cervical immune response. IL-12*α* and IL-18 are proinflammatory cytokines [155, 156] capable of inducing the production of interferon-*γ* (IFN-*γ*) [157] and cell-mediated immunity which are important factors in determining the progression of HPV related cervical lesion including cervical cancer [158]. Furthermore, the observed SF induction of cell adhesion molecules ICAM1 (3.82-fold induction) and ITGAM1 (4.78-fold induction) (Table 3) suggest that SF can also play a role in cervical cancer cell metastasis [159].

Taken together, regulation of these inflammatory pathways suggests that SF can play a critical role in cervical inflammation and consequently neoplastic transformation in the FMRT.

## 8. Conclusion

**Figure 1:**
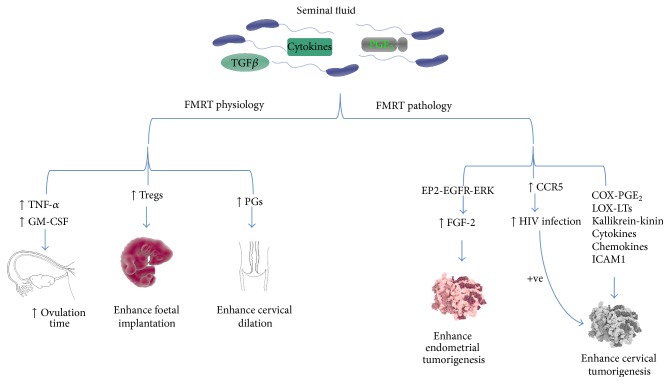
Schematic diagram showing the role of seminal fluid-mediated inflammation in the regulation of physiology and pathology of the female reproductive tract. SF has been shown to contain a wide variety of signaling molecules including cytokines, TGF*β*, and PGE_2_. Exposures of the female reproductive tract (FMRT) to these molecules can impact on the physiology and pathology of the FMRT. SF regulation of TNF-*α* and GM-CSF enhances ovulation process by increasing ovulation time. Regulation of Tregs population promotes maternal tolerance and foetal implantation while SF-mediated regulation of PGs enhances cervical dilation during parturition. It has been established that SF can mediate pathologic conditions within the FMRT. In endometrial tumorigenesis, SF-mediated regulation of FGF-2 via the activation of EP2-EGFR-ERK signaling enhances tumor progression. Similarly, by regulating COX-PGE_2_, LOX-LTs, kallikrein-kinin, cytokines, and chemokine signaling SF promotes cervical tumorigenesis in sexually active women. In addition, SF-mediated regulation of CCR5 (HIV chemokine coreceptor) further allude to its role in HIV infection and cervical tumorigenesis.

There is burgeoning body of* in vivo* and* in vitro* evidence for the involvement of SF-mediated inflammation in normal and pathophysiologic processes in the FMRT. SF has been shown to contain a wide variety of signaling molecules including cytokines, TGF*β*, and PGE_2_. Exposures of the female reproductive tract (FMRT) to these molecules can impact on the physiology and pathology of the FMRT. Differential expression of pleiotropic inflammatory cytokine genes as demonstrated by the TaqMan Plate Array data further gives credence to the fundamental role played by inflammatory mediators present in SF in regulating various pathways involved in physiologic and pathologic processes of the FMRT. These findings suggest that SF can potentially regulate the induction of inflammatory pathways in neoplastic cervix. Regulation of these inflammatory pathways plays a critical role in cancer cell angiogenesis, proliferation, invasion, metastasis, and survival. Overall, it is evident that the inflammatory response initiated by SF would impact on all physiological and pathophysiological events within the FMRT. As demonstrated schematically in Figure 1 and detailed in the figure legend, SF impact on normal physiology of the FMRT is on ovulation, implantation, and parturition. The pathological impact of SF is on promoting endometrial and cervical tumor growth and, in addition, increasing susceptibility to HIV infection. However, further research is clearly needed to elucidate in detail the mechanistic role of SF in FMRT physiology and pathology.

## Figures and Tables

**Table 1:** Components of eicosanoid signalling regulated by SF in the Taqman 96-well plate array. Results are expressed as fold above control.

Gene	Accession number	Fold change
*PTGS1*	NM_000962	2.43**↑**
*LTC4S*	NM_000897	10.03**↑**
*PLA2G2A*	NM_000300	3.00**↑**
*PLCB4*	NM_000933	2.82**↑**
*PLCG1*	NM_182811	2.90**↑**
*PLCE1*	NM_016341	6.71**↑**
*ANXA1*	NM_000700	6.66**↓**
*ANXA3*	NM_005139	3.44**↓**
*ANXA5*	NM_001154	>100**↓**
*HPGD*	NM_000860	3.12**↓**

↑: fold increase.

↓: fold decrease.

**Table 2:** Kallikrein-related peptidases regulated by SF in the Taqman 96-well plate array. Results are expressed as fold above control.

Gene	Accession number	Fold change
*KLK1*	NM_002257	1.56**↓**
*KLK2*	NM_001002231	19.59**↑**
*KLK3*	NM_001030047	3.45**↑**
*KLK14*	NM_022046	2.53**↑**
*KLKB1*	NM_000892	1.73**↑**

↑: fold increase.

↓: fold decrease.

**Table 3:** Proinflammatory cytokines/chemokines regulated by SF in the Taqman 96-well plate array. Results are expressed as fold above control.

Gene	Accession number	Fold change
*CCL2*	NM_002982	4.43**↑**
*CCL5*	NM_002985	5.24**↑**
*CXCL1*	NM_001511	12.37**↑**
*CXCL2*	NM_002089	2.04**↓**
*CXCL3*	NM_002090	8.19**↑**
*CXCL11*	NM_005409	2.47**↑**
*IL1A*	NM_000575	68.56**↑**
*IL6*	NM_000600	4.19**↑**
*IL8*	NM_000584	38.9**↑**
*IL12A*	NM_000882	2.36**↑**
*IL13*	NM_002188	1.39**↑**
*IL18*	NM_001562	17.84**↑**
*MYD88*	NM_002468	9.41**↑**
*NFKB1*	NM_001165412	2.32**↑**
*SOCS5*	NM_014011	>100**↓**
*TNF*	NM_000594	26.55**↑**
*TLR2*	NM_003264	88.47**↑**
*TLR4*	NM_138554	1.66**↑**
*ICAM1*	NM_000201	3.82**↑**
*ITGAM*	NM_000632	4.78**↑**

↑: fold increase.

↓: fold decrease.
